# The impact of authentic leadership on employees’ bootleg innovation behavior: the mediating role of affective commitment

**DOI:** 10.3389/fpsyg.2025.1581513

**Published:** 2025-09-04

**Authors:** Weiwei Liu, Mohamed Oubibi, Yunyun Xu, Bei Li

**Affiliations:** ^1^School of Management, Zhejiang University of Technology, Hangzhou, China; ^2^College of Education, Zhejiang Normal University, Jinhua, China; ^3^Faculty of Education, Smart Learning Institute of Beijing Normal University, Beijing Normal University, Beijing, China; ^4^School of Economics and Management, Zhejiang Normal University, Jinhua, China

**Keywords:** authentic leadership, bootleg innovation, affective commitment, self-awareness, relational transparency, internalized moral perspective, balanced processing.

## Abstract

**Purpose:**

According to the suggestions, the abstract has been revised: Innovation is the core competitiveness that maintains the continuous development of enterprises. Deviated innovation, as an important way for employees to engage in innovative activities and promote enterprises to enhance their competitive advantages, has become an increasingly important topic of concern in the academic circle. This study reveals the relationship between authentic leadership and employee bootleg innovation within the Chinese context, expanding research on the formation mechanisms of deviant innovative behaviors. It provides significant guidance for effectively leveraging authentic leadership, appropriately directing employee deviant innovative behaviors, and advancing enterprise management practices.

**Method:**

Based on social exchange theory and the cognitive-affective personality system theory, to explore the mechanisms through which authentic leadership influences employees’ deviant innovative behavior. The research focuses on high-tech enterprises in several provinces of China, collected 378 valid sample data of employees from high-tech enterprises. All data were analyzed using SPSS22.0, and the Bootstrap mediating effect test method was used to test the mediating effect results. The structural equation model was tested using AMOS24.0 statistical software.

**Results:**

Authentic leadership has a significant positive effect on deviant innovation behavior (β = 0.409, *P* < 0.001), and all four dimensions of authentic leadership have a significant positive impact on deviant innovation behavior. Self-awareness has a significant positive impact on employees’ deviant innovation behavior (β = 0.101, *P* < 0.05), relationship transparency has a significant positive impact on employees’ deviant innovation behavior (β = 0.196, *P* < 0.001), and internalized morality has a significant positive impact on employees’ deviant innovation behavior (β = 0.129). (*P* < 0.01), balanced information processing has a significant positive impact on employees’ deviant innovative behaviors (β = 0.268, *P* < 0.001). Authentic leadership has a positive effect on emotional commitment (β = 0.205, *P* < 0.001), and all four dimensions of authentic leadership have a significant positive impact on emotional commitment. Self-awareness has a significant positive impact on emotional commitment (β = 0.201, *P* < 0.001), relationship transparency has a significant positive impact on emotional commitment (β = 0.264, *P* < 0.001), and internalized morality has a significant positive impact on emotional commitment (β = 0.136, *P* < 0.01). Balanced information processing has a significant positive impact on emotional commitment (β = 0.361, *P* < 0.001). The mediating variable of affective commitment showed a considerable positive impact on bootleg innovation as well (β = 0.396, P < 0.001).

**Conclusion:**

The findings indicate that authentic leadership has a positive influence on employees’ deviant innovative behavior. Authentic leadership also positively affects affective commitment and affective commitment partially mediates the relationship between authentic leadership and employees’ deviant and innovative behavior. This study introduces an emotional perspective and takes emotional commitment as a mediating variable to explore the mediating transmission mechanism of emotional commitment between authentic leadership and employee deviant innovation. It enriches the research on the influence path of authentic leadership on the relationship of employee deviant innovation.

## Introduction

Innovation serves as a vital driving force for sustained economic growth and represents a core competitive advantage essential for the continuous development of enterprises. As key participants in enterprise innovation activities, employees’ creative ideas play a significant role in enhancing organizational competitiveness and innovative capacity. However, in practice, many employees’ innovative ideas remain unimplemented due to limited internal strategic resources within organizations and constraints imposed by external market environment changes. In an innovation-centric context, when organizations fail to fulfill employees’ desire for innovation, these individuals may resort to alternative means of engaging in bootleg innovation behavior. Bootleg innovation refers to behaviors employees undertake outside their formal roles, which contravene organizational norms but aim to enhance the organization’s overall well-being. When successful, such behaviors can yield remarkable innovative outcomes for the organization (Yang and Li, 2019). Research indicates that bootleg innovation is prevalent within organizations; over 80% of enterprises have reported experiencing instances of such behavior (Augsorfer, 2012). Furthermore, with the rapid development of information technology, especially after the outbreak of the COVID-19 pandemic, the popularization of online communication and remote working has provided more convenient resource conditions and organizational environments for employees to carry out deviant innovation. Compared with the past, remote positions, being far from the central organization and lacking direct supervision and management, allow employees to freely choose methods and flexibly arrange resources during the work process. This enhances employees’ confidence in their ability to implement innovative behaviors, thereby increasing their internal motivation to carry out deviant innovation and possibly making this phenomenon more and more common (Xiao, 2020; Globocnik et al., 2022). Consequently, bootleg innovation is increasingly acknowledged as a significant pathway for organizational advancement and has emerged as one of the key topics within academic discourse.

Since the concept of deviant innovation behavior was proposed, scholars have defined it in various ways, but all agree that the purpose of deviant innovation is to enhance organizational benefits and that it is a voluntary and autonomous behavior of employees (Criscuolo et al., 2014; Daft, 1978; Knight, 1967; Augsdorfer, 2005; Bledow et al., 2009; Lin et al., 2016; Jiang, 2018) defines it as a special form of innovation behavior that uses deviance as a means to achieve the goal of innovation. This paper mainly refers to the concept proposed by Criscuolo et al. (2014), that is, deviant innovation refers to the behavior of privately and spontaneously continuing to implement new ideas when individual innovation ideas are contrary to organizational management methods and norms, and the individual subjectively believes that this can improve the innovation performance of the organization. Deviant innovation behavior is a special form of innovation behavior, which is carried out in a “deviant” way and has two attributes: purpose legitimacy and behavioral deviation (Huang et al., 2017; Jiang, 2018). It is an innovative behavior activity aimed at improving organizational benefits but carried out in a way that deviates from organizational norms. Deviant innovation behavior is different from other behaviors such as constructive deviance. Both deviant innovation behavior and constructive deviance deviate from organizational norms and are behaviors initiated spontaneously by individuals to improve the overall benefits of the organization. However, deviant innovation behavior only includes the method of innovation, while constructive deviance involves a wider range and can be achieved through means other than innovation (Vadera et al., 2013). Although the results of deviant innovation behavior need to be discussed in specific contexts, to a certain extent, it reflects the innovation ability of employees and may become a source of continuous innovation for enterprises.

A review of relevant literature reveals that there are many factors influencing deviant innovation behavior, which can be mainly classified into two categories: organizational level and individual employee level. At the organizational level, the main factors include management processes, organizational atmosphere, and organizational resources. Management processes consist of process standardization (Mainemelis, 2010; Zhang and Oubibi, 2025) and proceduralized management models (Wang H. Y. et al., 2019). Organizational atmosphere encompasses organizational innovation atmosphere (Globocnik and Salomo, 2015), fairness atmosphere (Mainemelis, 2010), and friendly relationship atmosphere (Mainemelis, 2010). Organizational resources mainly refer to structural tension (Mainemelis, 2010). At the individual employee level, the main factors include individual characteristics, job characteristics, and willingness to take risks (Nora et al., 2011). Individual characteristics are further divided into the Five-Factor Model of personality (Uczuk, 2019) and proactive personality (Yang and Li, 2019). Job characteristics include creative self-efficacy (Jaiswal and Dhar, 2015) and job autonomy (Yang G. et al., 2019). Against the backdrop of the knowledge economy era, leadership factors play a crucial role in organizational development (Zhu et al., 2005), and the leadership style of leaders themselves has a profound impact on employees’ work attitudes, behaviors, and performance (Li and Zhang, 2021). According to social exchange theory, when leaders within an organization demonstrate respect for their employees and are willing to cultivate sincere and amicable relationships with them, they provide valuable resources for exchange. In return, employees are likely to enhance their work contributions and proactively engage in more positive behaviors, thereby maintaining and strengthening the relationship with their leaders. Previous studies have examined the effects of paternalistic leadership (Wang Y. F. et al., 2019) and transformational leadership (Wang H. Y. et al., 2019) on employees’ bootleg innovation behaviors. With the advancement of positive psychology and positive organizational behavior, both theoretical scholars and organizational managers have begun to focus on the impact of authentic leadership on employee behavior. Authentic leadership is characterized by positivity and openness, fostering a supportive environment within organizations. Authentic leadership style is more in line with the essence of positive leadership style and is conducive to stimulating employees’ innovative behavior. Furthermore, prior research has established that authentic leadership can promote employees’ innovative behaviors (Niu et al., 2018; Lin et al., 2022), creativity (Ribeiro et al., 2020), and work engagement (Ma et al., 2020). This raises an important question: Can authentic leadership encourage employees to engage in deviant innovative behaviors that benefit the organization? Currently, this issue remains under-explored by scholars.

Further literature review indicates that most prior studies have examined the relationship between leadership style and employees’ bootleg innovation behavior from a singular theoretical perspective, such as social cognition or social exchange. For instance, Wu S. J. et al. (2020) proposed from the perspective of social learning theory that authentic leadership positively influences employees’ deviant innovation behavior, with organizational self-esteem and constructive responsibility cognition playing a mediating role; Wang and Zhang (2019) suggested from the perspective of social cognitive theory that authentic leadership can enhance employees’ perception of their internal organizational identity, making them feel they are part of the organization, and thereby stimulating more innovative behaviors. These studies have validated the influence of organizational situational factors, individual cognition, and psychological elements on bootleg innovation; however, they have largely overlooked the role of emotional factors in stimulating employees’ deviant innovative behaviors. The cognitive-affective system of personality theory (CAPS) posits that leaders, as significant situational variables within organizations, can indirectly affect employee behavior by activating cognitive and affective units within individuals (Mischel and Shoda, 1995). Authentic leadership is characterized by its people-oriented and self-centered approach, embodying an informational content that emphasizes care for and respect toward employees. By fostering employees’ relational identification with leaders (cognitive unit) and enhancing their affective commitment to the organization (emotional unit), authentic leadership encourages employees to engage in deviant innovative behaviors that are beneficial for organizational development.

It is known from the literature that the existing theories of deviant innovation are mostly established in the Western context. However, the economic and cultural conditions of Chinese society, as well as the thinking and behavior of individuals, are different from those in the Western context, which may lead to certain differences in the manifestations of employees’ deviant innovation. For instance, there are different understandings of “face” in Chinese and Western cultures. As a typical cultural feature of Chinese society, “face” can have a significant impact on the behavior of employees in organizations (Tao et al., 2019). Additionally, the different cultural values in China and the West may also result in different deviant innovation behaviors. Chinese people are deeply influenced by Confucian culture, which advocates collectivism. Employees with a high sense of collectivism are more concerned about the welfare of the organization, which is in line with the attribute of deviant innovation that is oriented toward organizational interests, and thus may stimulate employees’ deviant innovation behaviors. Authentic leadership emphasizes moral concepts and adheres to the unity of internal morality and external behavior, which is highly consistent with the “morality” culture that is highly valued in Chinese society. Therefore, exploring the influence mechanism of authentic leadership on employees’ deviant innovation behaviors is of great significance for responding to the current scholars’ call for in-depth exploration of traditional Chinese culture and conducting management research based on the Chinese context, as well as improving the management practices of Chinese enterprises.

To summarize, this paper introduces authentic leadership as a situational factor influencing the formation mechanism of employees’ bootleg innovation behavior through the lens of social exchange theory. It also incorporates an emotional perspective grounded in cognitive-affective personality system theory, positioning affective commitment as a mediating variable to further investigate its role in authentic leadership and employees’ bootleg innovation. Based on the management context in China, this paper supplements the emotional path through which leadership style affects employees’ deviant innovation behavior on the basis of previous research, and explores more comprehensively the intrinsic relationship between authentic leadership and employees’ deviant innovation behavior. It further reveals the mechanism of the effect of authentic leadership on deviant innovation, and expands the research on the antecedents of employees’ deviant innovation behavior. The research results provide an important reference for leaders to scientifically deal with employees’ deviant innovation behavior.

## Literature review and research hypotheses

### The impact of authentic leadership on deviant innovation

In the prior review of literature concerning bootleg innovation, it was determined that leadership is perceived by the majority of scholars as a critical factor influencing employees’ engagement in bootleg innovation behaviors. The impact of authentic leadership on bootleg innovation manifests in four dimensions. First, authentic leaders exhibit openness to experience and possess intrinsic motivation; they pursue freedom and innovation while demonstrating strong self-awareness, effective self-management, and a positive emotional state (Tenzer and Yang, 2020; Oubibi, 2025). Inspired by these authentic leaders, employees are likely to be motivated by positive emotions and may even voluntarily engage in tasks that exceed organizational regulations, such as exhibiting deviant innovative behaviors. Secondly, authentic leaders align their actions with their own values and moral standards. They treat employees with sincerity and provide personal care while maintaining transparent relationships with them. This approach fosters a network of collaborative relationships (Hao and Cheng, 2015; Li, 2018). On one hand, enhancing employees’ psychological security and sense of belonging will enable them to focus on organizational objectives and actively pursue innovation, thereby improving overall organizational efficiency. On the other hand, it enhances leaders’ tolerance and support for employees’ constructive conflicts (Wang et al., 2012), which empowers employees to withstand the risks associated with innovation failures or opposition from leadership. This environment is conducive to promoting innovative behaviors that deviate from the norm (Wang and Zou, 2019). Furthermore, authentic leaders serve as role models whose distinctive behavioral traits positively influence employees’ psychological capital (Jiang, 2018). Employees are more inclined to persist when confronted with challenges, especially when their innovative endeavors are obstructed by organizational regulations and resource constraints. In such situations, they can maintain their courage and confidence while remaining true to their inner convictions, ultimately striving to implement innovation despite obstacles—resulting in deviant innovative behavior (Wu S. J. et al., 2020; Sun et al., 2025). Finally, authentic leaders demonstrate the capacity to respond to employee feedback in an objective and impartial manner. They do not dismiss, overlook, or evade issues related to voice behavior simply because these matters may involve personal interests (Li X. et al., 2016). Instead, they actively respond to employees’ needs for work autonomy, thereby creating an environment that fosters space for bootleg innovation behaviors (Wu Y. M. et al., 2020).

The social exchange theory posits that when individuals perceive they have received certain benefits, they are likely to take proactive measures to reciprocate. Authentic leadership conveys positive emotions to employees and is capable of providing objective evaluations. Such leaders demonstrate care for their subordinates, strive to establish open relationships with them and engage in equitable communication. These behaviors contribute significantly to enhancing the trust relationship between leaders and employees. In return for the sincerity and trust exhibited by their leaders, employees are inclined to engage in extra-role behaviors for the benefit of the organization; they may even be willing to assume certain risks by adopting informal approaches to pursue innovative deviance when organizational resources are limited. Based on this premise, this paper proposes the following research hypothesis:

H1: There is a positive correlation between authentic leadership and employees’ engagement in bootleg innovation behavior.

H1a: There is a positive correlation between self-awareness and the bootleg innovation behavior exhibited by employees.

H1b: There is a positive correlation between the internalized moral perspective and employees’ bootleg innovation behavior.

H1c: Relationship transparency is positively associated with employees’ bootleg innovation behavior.

H1d: Balanced processing is positively associated with employees’ bootleg innovation behavior.

### The influence of authentic leadership on emotional commitment

Now, several scholars have demonstrated that leadership behavior significantly influences employees’ organizational (emotional) commitment. Avolio et al. were the first to identify the relationship between authentic leadership and employees’ organizational commitment, theoretically predicting in 2004 that authentic leadership correlates with organizational commitment (Walumbwa et al., 2008). The study revealed that quiet leadership has a positive impact on job performance, with organizational commitment acting as a partial mediator (Li J. et al., 2016). They also identified leaders’ humility as a catalyst for affective commitment, restraint as a driver of behavioral commitment, and persistence as a promoter of loyalty commitment, thereby yielding substantial practical implications for job performance (Li J. et al., 2016). Park and Seo (2019) demonstrated that shared leadership has a positive effect on organizational commitment, mediated by psychological empowerment and perceptions of organizational justice. Liu and Kong highlighted that transformational leadership exhibited by principals has significant positive effects on teachers’ self-efficacy across three dimensions as well as their organizational commitment (Liu and Kong, 2020). Leroy et al. discovered that both authentic leadership and behavioral integrity are key drivers of employees’ emotional organizational commitment and job performance (Leroy et al., 2012). According to Oh J. and Oh S., authentic leadership exerts a negative indirect effect on employees’ turnover intentions through variations in levels of affective commitment (Oh and Oh, 2017).

Specifically, due to their inherent characteristics, authentic leaders exert influence on affective commitment in four key aspects. Firstly, authentic leaders possess a strong sense of self-awareness and engage in self-assessment while encouraging employees to express themselves freely (Han and Liu, 2020). This approach enhances employee participation within the organization. Through self-assessment, leaders can identify their strengths and weaknesses as well as recognize their impact on others through interpersonal interactions. Such awareness is likely to mitigate personal biases. Consequently, employees who feel empowered to communicate openly will experience a sense of trust and respect from their leaders, leading them to become more engaged in organizational activities and thereby strengthening their affective commitment to the organization. Secondly, authentic leaders have internalized moral values, and their equitable leadership style aligns with the moral standards and intrinsic values of the majority of employees (Li et al., 2014). These leaders regulate their behavior according to internal moral principles, demonstrating high levels of integrity in their interactions with employees. As a result, they are more readily recognized and accepted by staff members, fostering greater affective commitment to the organization among employees. Furthermore, the relationship between authentic leaders and employees is characterized by transparency, which enhances employees’ sense of belonging and security (Wang and Zhang, 2019). Authentic leaders actively cultivate an atmosphere of transparent organizational relationships that mitigates mutual suspicion while strengthening trust and emotional attachment within the workforce. Finally, authentic leaders actively seek diverse opinions and engage in balanced discussions before making decisions. This approach fosters a sense of respect among employees (Liang et al., 2016), leading them to feel that their identities are acknowledged and valued by the organization. Consequently, this recognition generates increased emotional loyalty toward the organization.

Based on these observations, this paper proposes the following hypothesis for further research:

H2: Authentic leadership is positively correlated with affective commitment.

H2a: Self-awareness is positively correlated with affective commitment.

H2b: Internalizing moral values is positively correlated with affective commitment.

H2c: Relationship transparency is positively correlated with affective commitment.

H2d: Balanced processing is positively correlated with affective commitment.

### The impact of emotional commitment on deviant innovation

Previous research has demonstrated that affective commitment plays a significant role in influencing innovative behavior. For instance, Ma and Su (2020) demonstrated that individuals with affective commitment to an organization are more likely to engage in innovation-related activities. Additionally, Xiao (2020) confirmed that for remote employees exhibiting high organizational commitment, the positive effect of job autonomy on bootleg innovation behavior is amplified. The findings can be summarized in three key aspects. First and foremost, continuous internal motivation serves as a prerequisite for employees to innovate. This internal motivation encourages employees to focus more on the complexity and innovative aspects of their work, fostering a willingness to take risks and enhancing their inclination toward creative problem-solving (Ma and Su, 2020). Employees who exhibit a strong affective commitment to their organization possess one of the key internal motivations for innovation—namely, finding enjoyment in their work. Such employees typically derive intrinsic satisfaction from the nature of their tasks and are more inclined to proactively engage in innovative activities that may deviate from established norms. Furthermore, individuals with a robust emotional attachment to the organization tend to experience a heightened sense of identity and belonging (Ma and Su, 2020). They often perceive it as their duty to contribute to the organization’s development and aspire it to maintain a leading competitive position, which consequently leads them to demonstrate more positive behaviors (Leroy et al., 2012). Yan and Zhang (2017) also noted that when employees possess a strong identification with the organization and actively participate in their roles, innovative behaviors can be anticipated. Lastly, employees characterized by high levels of affective commitment generally have broader role perceptions. This allows them to incorporate out-of-role behaviors into their responsibilities more readily (Ma and Su, 2020). Motivated by a sense of responsibility, they are likely to adopt more effective strategies for task completion; thus, they are predisposed toward generating out-of-role innovative behaviors that benefit the organization.

Based on these observations, this paper proposes the following hypothesis for further research:

H3: There is a positive correlation between affective commitment and bootleg innovation.

### The mediating role of emotional commitment

Although there are limited empirical studies examining the mediating role of affective commitment between authentic leadership and bootleg innovation in China, some scholars have validated the mediating effect of affective commitment on other employee outcomes. For instance, Ribeiro et al. (2020) demonstrated through empirical research that affective commitment serves as a complete mediator between perceived authentic leadership and individual creativity. Similarly, Li et al. (2014) confirmed the mediating role of affective commitment between authentic leadership and job engagement. These findings suggest that authentic leadership can influence employee behavior by enhancing their affective commitment. When leaders delegate certain decision-making authority to employees and share responsibilities with them, it cultivates an environment that promotes the enhancement of employees’ emotional commitment. (Ribeiro et al., 2019). In the workplace, employees who possess strong emotional ties to their organization tend to exhibit greater identification with it and demonstrate enhanced creativity; they are also more likely to approach work tasks proactively and innovatively (Ma and Wang, 2016). Authentic leaders effectively stimulate employees’ affective commitment due to their heightened self-awareness, consistent behaviors, transparent cooperative relationships with staff members, and equitable handling of feedback from employees. Employees motivated by this sense of affective commitment are more likely to express intrinsic motivation, willingness to take risks and innovation potential—all factors that facilitate the emergence of deviant innovative behaviors.

At the same time, drawing on the cognitive-affective personality system theory, the activated affective unit of employees emerges as a significant factor influencing their behaviors. When employees’ affective commitment is stimulated, they are more inclined to exhibit deviant innovative behaviors (Mischel and Shoda, 1995). Employees with high levels of affective commitment possess profound feelings for the organization that extends beyond mere self-interest; they are eager to invest in organizational development and consciously protect both its interests and image (Wang Y. F. et al., 2019). Even when faced with potential risks associated with bootleg innovation, their emotional attachment to the organization motivates them to undertake work tasks that exceed their defined responsibilities. Consequently, employees exhibiting high affective commitment tend to prioritize the realization of overarching organizational interests. They persistently advocate for original innovative ideas and demonstrate an increased propensity for engaging in deviant innovative behaviors that benefit the organization. Based on this understanding, this study proposes the following hypothesis:

H4: Affective commitment plays a mediating role between authentic leadership and bootleg innovation behavior.

### Theoretical model

**FIGURE 1:**
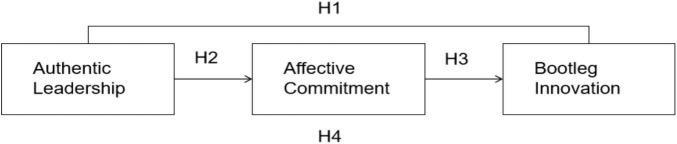
Theoretical model supplementary tables.

Based on the aforementioned research hypotheses, this paper constructs a research model that examines the relationships among authentic leadership, affective commitment, and bootleg innovation. In this model, the four dimensions of authentic leadership: self-awareness, internalized moral perspective, relationship transparency, and balanced processing are treated as independent variables. Affective commitment serves as a mediating variable, while bootleg innovation is regarded as the dependent variable. This framework is illustrated in Figure 1.

## Questionnaire design and data collection

### Questionnaire design

This paper employs a questionnaire-based approach for data collection. The scale of the three selected variables has been widely applied and recognized among scholars at home and abroad. In light of this, the paper has refined the relevant descriptions by the actual research context to develop a comprehensive scale questionnaire. The designed questionnaire comprises four distinct sections:

The initial section comprises a description of the questionnaire along with fundamental personal information. This includes the participant’s gender, age, educational background, job title, occupational category, years of experience in their field, and six additional items.

The second part of the questionnaire is the measurement of authentic leadership. Since the concept of authentic leadership was proposed, its connotation has been constantly evolving. Different researchers have different understandings of the connotation of authentic leadership, so they have different divisions of the dimensions of authentic leadership. Given that Walumbwa’s scale was initially tested with Chinese data during its development, and considering that numerous scholars (Wu S. J. et al., 2020; Han and Liu, 2020; Ma et al., 2020; Li, 2018; Yang J. et al., 2019; Wang and Zhang, 2019) have validated and utilized it within China, this scale is deemed appropriate for adoption in this paper due to its high level of maturity. The scale categorizes authentic leadership into four dimensions: self-awareness, internalized morality, transparent relationships, and Balanced processing, comprising a total of 16 items. Self-awareness primarily pertains to an individual’s worldview and their understanding of personal strengths and weaknesses. It involves ongoing reflection on one’s self-concept as well as an awareness of how one impacts others based on feedback received during interpersonal interactions. Internalizing ethics mainly refers to the process of self-regulation through the internalization of moral standards and values. Transparent relationships emphasize leaders’ authenticity in presenting their true selves while establishing credible and open connections with employees. Finally, Balanced processing entails analyzing all relevant data objectively without distorting facts before making decisions.

The third part addresses the measurement of affective commitment. emotional commitment. As an important dimension of organizational commitment, the scale of emotional commitment mostly adopts some items from the organizational commitment scale. There are two most representative scales. The first one was the scale of organizational commitment designed by Porter in 1974, which had 15 items. Among the collected literature, few scholars have adopted this scale. Secondly, there is the organizational commitment scale designed by Meyer and Allen. Initially, the part of the scale that belongs to emotional commitment had 8 items. Later, they modified the number of items from 8 to 6. Among the literature reviewed, only three articles utilized Porter’s scale, while the majority employed Meyer and Allen’s scale. This indicates that Meyer and Allen’s scales are currently recognized and widely adopted within the academic community, with numerous Chinese scholars contributing to their application (Ma and Su, 2020; Li, 2018; Su, 2017; Zhu et al., 2016; Ma and Wang, 2016), particularly in conjunction with background verification relevant to China. Consequently, this paper will adopt Meyer and Allen’s six-item scale for its analysis.

The fourth part addresses the measurement of bootleg innovation. For the measurement of deviant innovation, there are mainly two schools of thought, Lin and Criscuolo. Other than that, there are some scattered scholars. Currently, the measurement is basically from a single dimension. In mainstream research, Lin’s scale necessitates assessment within 2 months following the occurrence of bootleg innovation; however, it poses challenges in determining whether the subjects’ instances of bootleg innovation fall within this timeframe. Consequently, this paper will adopt an alternative mainstream instrument: the 5-item scale developed by Criscuolo. This scale has also been validated by numerous Chinese scholars (Wang H. Y. et al., 2019; Guo, 2020; Xiao, 2020; Zhao et al., 2019; Li et al., 2019; Yang and Li, 2019) in conjunction with a Chinese contextual background verification. All items in the questionnaire were rated on a Likert-5 scale, where scores ranging from 1 to 5 correspond to “very inconsistent” and “very consistent,” respectively.

## Data collection

This research is grounded in three theoretical frameworks: authentic leadership, affective commitment, and bootleg innovation. To ensure that the research samples are both applicable and representative, this study draws upon Wu S. J. et al. (2020) findings. On one hand, the subjects of this research comprise employees from high-tech industries that necessitate frequent innovation, such as information technology, mechanical manufacturing, and biomedicine. On the other hand, the selected employee categories include both regular staff members and managerial personnel. This selection is based on the premise that these employees are more likely to exercise autonomy in their work task arrangements, thereby enhancing their capacity to identify business opportunities.

The sample range of this study encompasses Beijing, Shenzhen, Hangzhou, Ningbo, and Wenzhou in Zhejiang Province. These regions are characterized by rapid economic development, with enterprises placing a strong emphasis on innovation; consequently, their innovation systems surpass the national average. The survey was conducted using a combination of electronic questionnaires and offline paper questionnaires. To minimize errors and enhance the accuracy and authenticity of the responses, participants were encouraged to complete the questionnaire in a relaxed manner. Before distribution, the purpose and process of the survey were explained to human resources department heads within participating enterprises for overall coordination. The online questionnaire was primarily distributed to companies located in Beijing and Shenzhen due to logistical challenges associated with conducting field investigations in these areas. A total of 473 questionnaires were collected through both online and offline methods; after screening for validity—eliminating those with incorrect answers, missing responses, or logical inconsistencies—378 valid questionnaires remained.

### Empirical analysis

This section uses SPSS22.0 and AMOS24.0 software to conduct statistical analysis on the collected data and empirically test the proposed hypotheses. The main steps include: descriptive statistics, reliability and validity analysis of the scale, correlation analysis, common method variance test, structural equation model test, and mediating effect test. Among them, the preliminary analysis includes descriptive statistics, reliability and validity analysis of scales, correlation analysis and common method variance test. Descriptive analysis serves two purposes: one is to understand the basic characteristics of the sample, and the other is to check whether the scale data conforms to a normal distribution, providing fundamental information for subsequent analysis. The reliability and validity test of the scale is to verify whether the questionnaire scale is scientific and effective and whether it can effectively measure the scale. Correlation analysis is to test whether there is a correlation between variables, while the common method variance test is to detect whether the data is systematically biased due to a single measurement method and avoid misjudging the true relationship between variables. These preliminary tests lay the foundation for the subsequent structural equation model analysis and mediating effect analysis.

### Descriptive statistics of the sample

Firstly, descriptive statistics of the sample are conducted. One is to perform frequency statistics on the information contained in the sample, such as gender, age and other characteristics. The other is to analyze the basic level of the items in the scale and the distribution of data presentation.

## Statistical analysis of basic information frequency

In this study, frequency statistics were conducted on basic demographic information, including gender, age, educational background, position, job category, and years of work experience. The percentage of each option relative to the total sample size was calculated. The results are presented in Table 1. It has been noted that males comprised 58.5% of the total respondents; this distribution may be affected by the characteristics of the selected sample enterprises. The predominant age groups are those under 25 years old and between 26–30 years old, accounting for 33.1 and 40.2%, respectively; this trend may also reflect the age structure typical of employees within high-tech industries. Regarding educational background, a significant majority of participants were undergraduates (45.8%), while individuals holding master’s degrees or higher comprised 22.2%. This suggests that most subjects possess a commendable level of education and exhibit a high degree of objectivity in their responses. The majority held positions as ordinary employees; however, when combined with various managerial roles, these accounted for approximately 50% of all positions surveyed. The primary job categories identified were production and technology-related roles. Finally, it was found that most respondents had been working for a duration primarily ranging from 2–5 years.

**TABLE 1:** Basic information frequency statistics.

Items	Categories	Frequency	Percentage (%)
Gender	Male	221	58.5
	Female	157	41.5
Age	Age 25 and under	125	33.1
	Ages 26–30	152	40.2
	31–40 years old	87	23.0
	41–50 years old	13	3.4
	Age 51 and older	1	0.3
Education	College degree or less	121	32.0
	Bachelor’s degree	173	45.8
	Master’s degree or above	84	22.2
Positions	Rank and file	189	50.0
	Lower-level managers	92	24.3
	Middle managers	70	18.5
	Top managers	27	7.1
Job category	Management (administration, personnel, finance)	65	17.2
	Technical research and development	112	29.6
	Production	135	35.7
	Sales category	66	17.5
Years of work	Less than 1 year	101	26.7
	2–5 years	180	47.6
	6–10 years	75	19.8
	10 + years	22	5.8
Total	378	100.0

## Descriptive statistical analysis

This paper conducts descriptive statistics on the Authentic Leadership Scale (items AL1-AL16), the Affective Commitment Scale (items AC1-AC6), and the Deviance Innovation Scale (items BI1-BI5). The aim is to assess the fundamental levels of the items within these scales and to analyze the distribution of data presentation.

Table 2 presents the results of the statistical analysis conducted on the data obtained from the questionnaire. Descriptive statistical analysis of the scale is to determine whether the data conforms to a normal distribution and provide a basis for subsequent analysis. Including the minimum value, maximum value, skewness and kurtosis value. The mean is the arithmetic mean of all values in a dataset, reflecting the central trend of the data. The standard deviation is an indicator of the degree of data dispersion (fluctuation size). The maximum and minimum values provide the upper and lower limits of the data range, reflecting the fluctuation range of the data. As can be seen from the table, the maximum value of the number is 5, the minimum value is 1, the mean value is between 3.7 and 3.9, and the standard deviation is between 0.8 and 1.1. The data is relatively concentrated and fluctuates little. In addition to these three indicators, the most important ones are skewness and kurtosis. According to literature review, when the absolute value of skewness is less than 3 and the absolute value of kurtosis is less than 10, it indicates that the measurement data basically conform to the normal distribution and can proceed to the next step of analysis (Kline, 1998). The absolute value of the maximum skew in this study is 1.08 < 3, and the absolute value of the kurtosis is 1.26 < 10. It is indicated that each question can follow a normal distribution. The data collected from the questionnaire can be directly used for subsequent statistical analyses such as reliability and validity.

**TABLE 2:** Descriptive statistics.

Measures	N	Min	Max	M	SD	S	K
AL1	378	1.00	5.00	3.844	1.090	−0.997	0.447
AL2	378	1.00	5.00	3.780	1.005	−0.683	−0.014
AL3	378	1.00	5.00	3.857	0.956	−0.719	0.063
AL4	378	1.00	5.00	3.852	0.980	−0.874	0.652
AL5	378	1.00	5.00	3.847	0.971	−0.791	0.230
AL6	378	1.00	5.00	3.905	0.893	−0.823	0.741
AL7	378	1.00	5.00	3.997	0.869	−0.798	0.404
AL8	378	1.00	5.00	3.825	0.967	−0.759	0.267
AL9	378	1.00	5.00	3.868	0.982	−0.899	0.547
AL10	378	1.00	5.00	3.905	0.962	−0.888	0.545
AL11	378	1.00	5.00	3.944	0.938	−0.819	0.338
AL12	378	1.00	5.00	3.947	0.908	−0.899	0.785
AL13	378	1.00	5.00	3.685	1.042	−0.684	−0.032
AL14	378	1.00	5.00	3.886	0.988	−0.865	0.261
AL15	378	1.00	5.00	3.841	0.959	−0.749	0.238
AL16	378	1.00	5.00	3.794	1.030	−0.706	0.000
AC1	378	1.00	5.00	3.862	0.994	−1.055	1.085
AC2	378	1.00	5.00	3.717	0.992	−0.737	0.184
AC3	378	1.00	5.00	3.751	1.101	−0.839	0.040
AC4	378	1.00	5.00	3.844	1.006	−1.080	0.995
AC5	378	1.00	5.00	3.857	0.996	−0.956	0.720
AC6	378	1.00	5.00	3.849	1.033	−0.855	0.152
BI1	378	1.00	5.00	3.907	0.901	−0.934	1.084
BI2	378	1.00	5.00	3.873	0.966	−0.701	−0.019
BI3	378	1.00	5.00	3.937	0.872	−0.939	1.260
BI4	378	1.00	5.00	3.873	0.988	−0.838	0.282
BI5	378	1.00	5.00	3.860	0.946	−0.906	0.726

## Reliability analysis of the scale

Reliability analysis primarily assesses the correlation, consistency, and stability of each item within the scale. This analysis determines whether the scale is reliable and evaluates potential deviations in responses from different subjects across various environments. Cronbach’s Alpha coefficient is commonly employed for this testing. The value of Cronbach’s Alpha ranges from 0 to 1 (Sun, 1998). Generally, a coefficient greater than 0.7 indicates that the recovered sample data possesses high reliability; when the coefficient falls between 0.35 and 0.7, it suggests that reliability is within an acceptable range; conversely, if the coefficient is below 0.35, it signifies poor reliability of the questionnaire, indicating a need to reconsider or revise the scale design.

Variables are primarily reduced to enhance reliability, which is generally assessed based on two criteria: First, if the Corrected Item-Total Correlation (CITC) of a deleted item is below 0.5, that item will be removed; second if the Cronbach’s Alpha coefficient increases following the deletion of an item, that item will also be eliminated. This study utilizes these two criteria as the foundation for item purification. The overall reliability of the questionnaire employed in this research is presented in Table 3, while the scale reliability test results for the three variables examined are displayed in Table 3.

**TABLE 3:** Results of scale reliability test.

Variables	Dimensions	Item	CITC	Item deletion Cronbach’s alpha	Cronbach’s alpha	
AL	SA	AL1	0.686	0.808	0.848	0.849
		AL2	0.730	0.787		
		AL3	0.667	0.814		
		AL4	0.662	0.816		
	RT	AL5	0.823	0.825	0.879	
		AL6	0.683	0.860		
		AL7	0.668	0.863		
		AL8	0.686	0.859		
		AL9	0.702	0.856		
	IMP	AL10	0.672	0.822	0.853	
		AL11	0.702	0.810		
		AL12	0.745	0.793		
		AL13	0.666	0.828		
	BP	AL14	0.691	0.681	0.801	
		AL15	0.577	0.798		
		AL16	0.676	0.697		
AC		AC1	0.681	0.876	0.891	
		AC2	0.790	0.859		
		AC3	0.664	0.880		
		AC4	0.708	0.872		
		AC5	0.653	0.880		
		AC6	0.765	0.863		
BI		BI1	0.705	0.860	0.882	
		BI2	0.788	0.840		
		BI3	0.671	0.867		
		BI4	0.706	0.860		
		BI5	0.719	0.856		

The results show the overall coefficient of the questionnaire is 0.923, which is much higher than 0.7, indicating that the overall reliability of the questionnaire is high.

As illustrated in the Table above, Cronbach’s Alpha for authentic leadership (AL) examined in this study is 0.849. The Cronbach’s Alpha values for self-awareness (SA), relationship transparency (RT), internalized moral perspective (IMP), and balanced processing (BP) are 0.848, 0.879, 0.853, and 0.801 respectively; all of these exceed the threshold of 0.7. Furthermore, Cronbach’s Alpha for affective commitment is recorded at 0.891, while that for bootleg innovation (BI) stands at 0.882. Since the coefficient for each variable exceeds 0.7, it indicates that each variable demonstrates good reliability. Additionally, the Corrected Item-Total Correlation (CITC) for each question is greater than 0.5; moreover, an analysis from the perspective of “deleting this item’s Cronbach’s Alpha value” reveals that removing any individual question would not increase Cronbach’s Alpha value. Therefore, it is recommended to retain all items within each variable.

### Validity analysis of the scale

Validity analysis serves as an index for assessing the extent to which our measurement tools accurately reflect the target data, commonly referred to as validity. In essence, a higher level of validity corresponds to greater accuracy. Factor analysis is a widely employed method for conducting validity assessments. This paper utilizes both exploratory factor analysis and confirmatory factor analysis to evaluate validity.

### Exploratory factor analysis

Generally, two prerequisites must be met for conducting exploratory factor analysis: first, the KMO (Kaiser-Meyer-Olkin) value should exceed 0.6; second, the significance level (Sig) of Bartlett’s test of sphericity must be less than 0.05. Consequently, both KMO and Bartlett tests will be conducted before performing exploratory factor analysis in this study. The following conducts exploratory factor analyses on the three variables of authentic leadership, emotional commitment, and deviant innovation respectively.

First, KMO and Bartlett tests are conducted for authentic leadership, and the analysis results are shown in Table 4.

**TABLE 4:** KMO and Bartlett tests of authentic leaders.

Kaiser-Meyer-Olkin measure of sampling adequacy		0.837
Bartlett’s sphericity test	Approximate Chi-square	2836.800
	Degrees of freedom	120
	Significance	0.000

As illustrated in Table 5, the KMO value for authentic leadership was 0.837, which exceeds the threshold of 0.6. The approximate chi-square statistic from Bartlett’s test was 2836.800, with a degree of freedom of 120 and a significance level of 0.000, which is less than the critical value of 0.05. These results satisfy both conditions necessary for conducting factor analysis. Consequently, this study employed principal component analysis along with an orthogonal rotation method (refer to Table 6 for detailed results) to extract four factors that exhibited eigenvalues greater than one. The cumulative variance explained by these four factors amounted to 69.432%, indicating a robust explanatory power regarding their interrelationships. Furthermore, all factor loadings post-rotation were above the threshold of 0.5, and no cross-loadings were observed among the factors. Overall, these findings suggest that the construct validity of the authentic leadership scale is commendable.

**TABLE 5:** Component matrix and total variance interpretation of authentic leadership.

Dimensions	Item	Ingredients			
		1	2	3	4
SA	AL1			0.811	
	AL2			0.849	
	AL3			0.804	
	AL4			0.799	
RT	AL5	0.877			
	AL6	0.784			
	AL7	0.769			
	AL8	0.771			
	AL9	0.801			
IMP	AL10		0.789		
	AL11		0.827		
	AL12		0.867		
	AL13		0.793		
BP	AL14				0.849
	AL15				0.769
	AL16				0.849
Rotation sums of squared loading	Total	3.381	2.802	2.765	2.161
	variance%	21.129	17.511	17.283	13.509
	Cumulative%	21.129	38.640	55.923	69.432

**TABLE 6:** KMO and Bartlett tests of affective commitment.

Kaiser-Meyer-Olkin measure of sampling adequacy		0.898
Bartlett’s sphericity test	Approximate Chi-square	1164.005
	Degrees of freedom	15
	Salience	0.000

Second, KMO and Bartlett tests are conducted on affective commitment, and the analysis results are shown in Table 6.

As illustrated in Table 6, the KMO value for affective commitment is 0.898, which exceeds the threshold of 0.6. The approximate chi-square statistic from Bartlett’s test is 1164.005, with a degree of freedom of 15 and a significance level of 0.000, which is less than the critical value of 0.05. These results satisfy both conditions necessary for conducting factor analysis. Consequently, this study employed principal component analysis along with an orthogonal rotation method to examine affective commitment (as presented in Table 7). A single factor was extracted that possesses an eigenvalue greater than 1, and this factor accounts for a cumulative variance explanation rate of 64.949%, indicating robust explanatory power regarding the variance associated with it. Furthermore, all load coefficients for the factors after rotation are above the threshold of 0.5, suggesting that the affective commitment scale demonstrates strong construct validity overall.

**TABLE 7:** Component matrix and total variance interpretation of affective commitment.

Dimensions	Item	Ingredients
		1
AC	AC1	0.783
	AC2	0.866
	AC3	0.769
	AC4	0.806
	AC5	0.760
	AC6	0.847
Extraction sums of squared loading	Total	3.897
	variance%	64.949
	Cumulative%	64.949

Third, KMO and Bartlett tests are carried out on bootleg innovation, and the analysis results are shown in Table 8.

**TABLE 8:** KMO and Bartlett tests of bootleg innovation.

Kaiser-Meyer-Olkin measure of adequacy of sample		0.876
Bartlett’s sphericity test	Approximate Chi-square	945.613
	Degrees of freedom	10
	Salience	0.000

As illustrated in Table 9, the KMO value for bootleg innovation is 0.876, which exceeds the threshold of 0.6. The approximate chi-square statistic from the Bartlett test is 945.613, with a degree of freedom of 10 and a significance level of 0.000, which is less than the critical value of 0.05; thus, both conditions for conducting factor analysis are satisfied. Consequently, this study employed principal component analysis along with an orthogonal rotation method for bootleg innovation (as presented in Table 10), extracting factors with eigenvalues greater than one. The cumulative variance explained by this factor was found to be 68.042%, indicating a strong explanatory power regarding the variance associated with this factor. Furthermore, all rotated factor load coefficients exceeded the threshold of 0.5. In summary, the framework validity of the bootleg innovation scale demonstrates robust quality.

**TABLE 9:** Component matrix and total variance interpretation of bootleg innovation.

Dimensions	Item	Ingredients
		1
BI	BI1	0.816
	BI2	0.875
	BI3	0.789
	BI4	0.816
	BI5	0.825
Extraction sums of squared loading	Total	3.402
	variance%	68.042
	Cumulative%	68.042

**TABLE 10:** Analysis and test of structural validity of each scale.

Variable	Fitting index	χ^2^/df	RMSEA	GFI	TLI	CFI
	Standard value	<3	<0.08	>0.90	>0.90	>0.90
AL	Actual value	1.843	0.047	0.943	0.963	0.970
AC	Actual value	2.480	0.063	0.982	0.981	0.988
BI	Actual value	2.068	0.053	0.989	0.989	0.994
Overall	Actual value	1.309	0.029	0.927	0.980	0.982

### Confirmatory factor analysis

In this paper, confirmatory factor analysis is conducted from three perspectives: structural validity, convergent validity, and discriminative validity. The analysis of discriminative validity is further examined in conjunction with correlation analysis in the subsequent sections of the paper.

**FIGURE 2:**
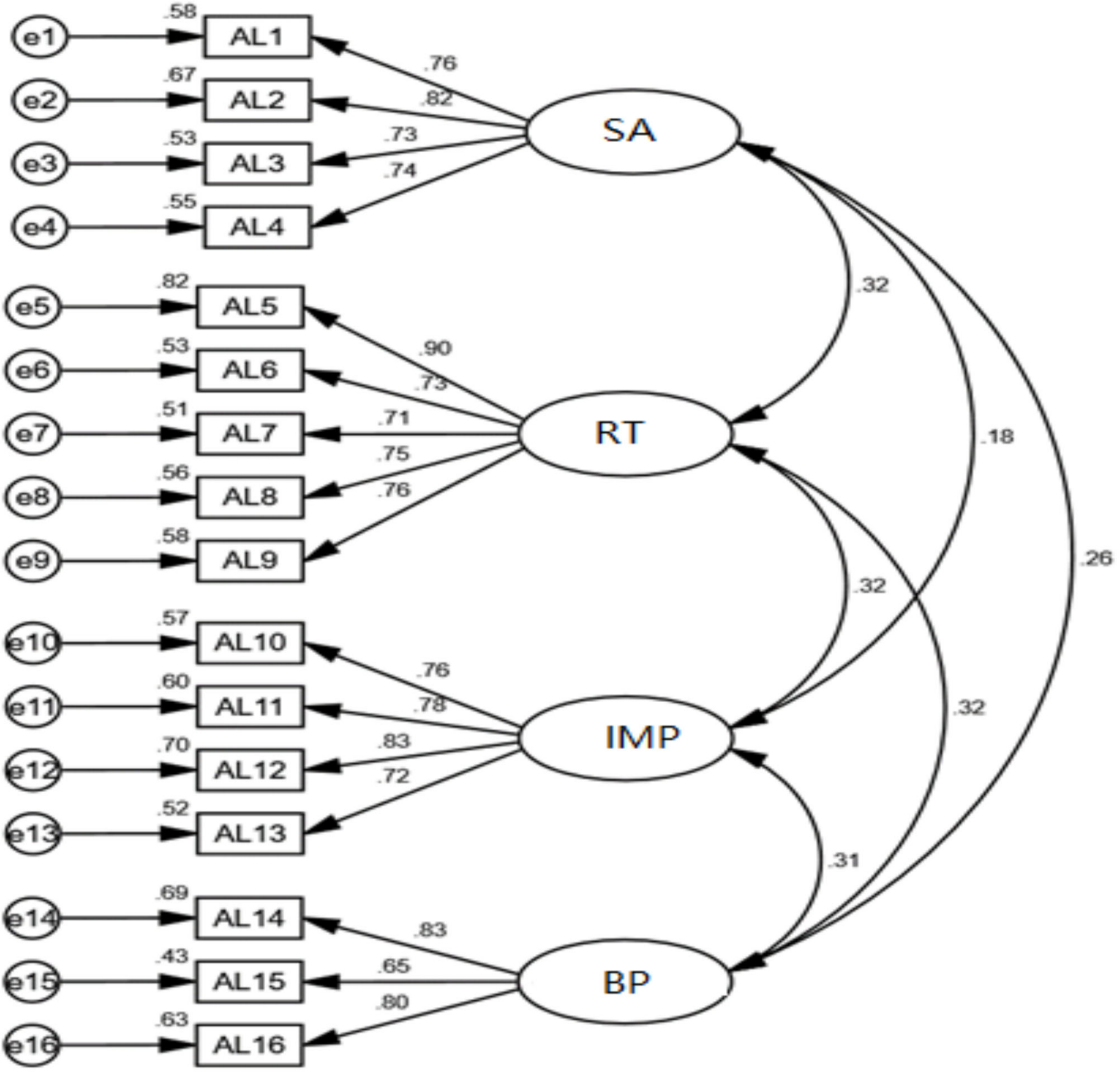
Confirmatory factor analysis diagram of authentic leadership.

**FIGURE 3:**
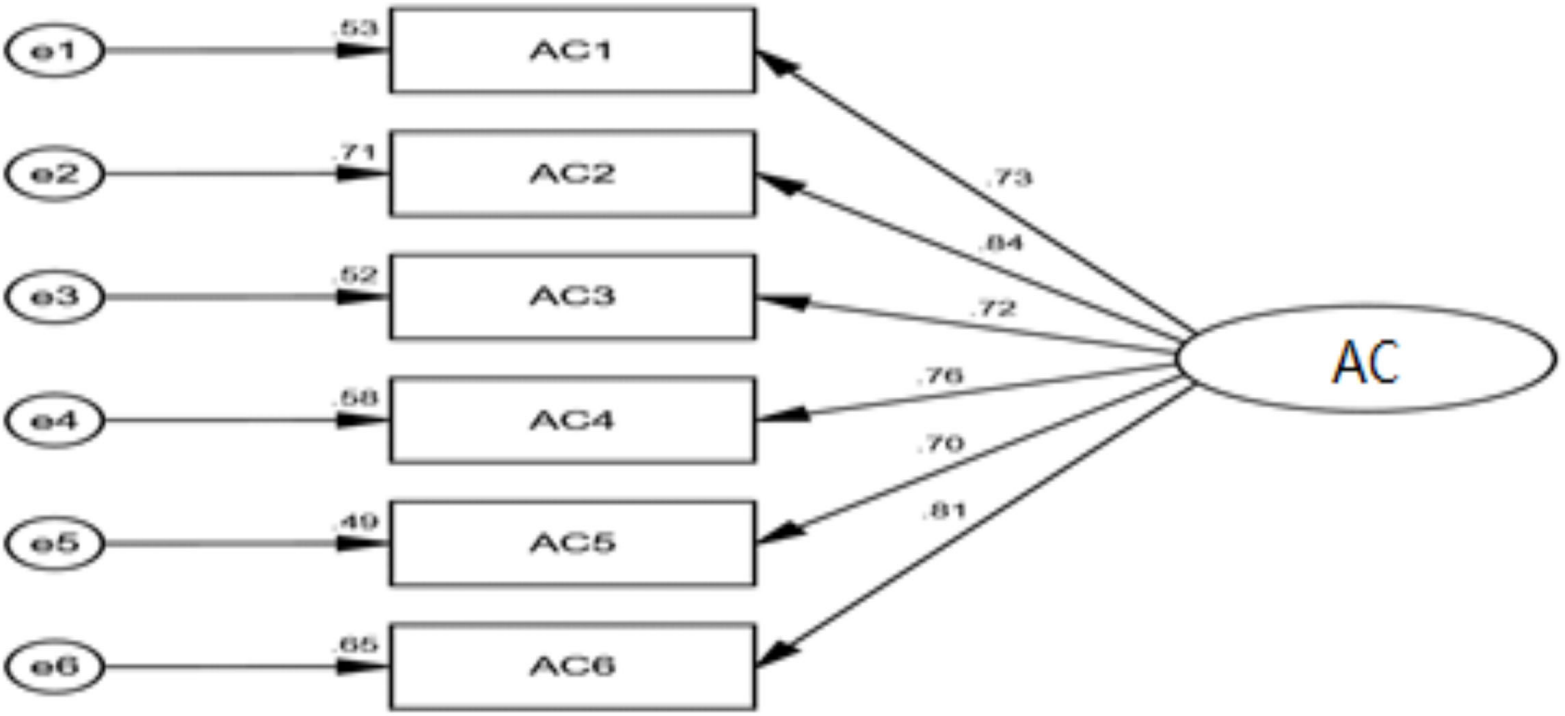
Confirmatory factor analysis diagram of affective commitment.

**FIGURE 4:**
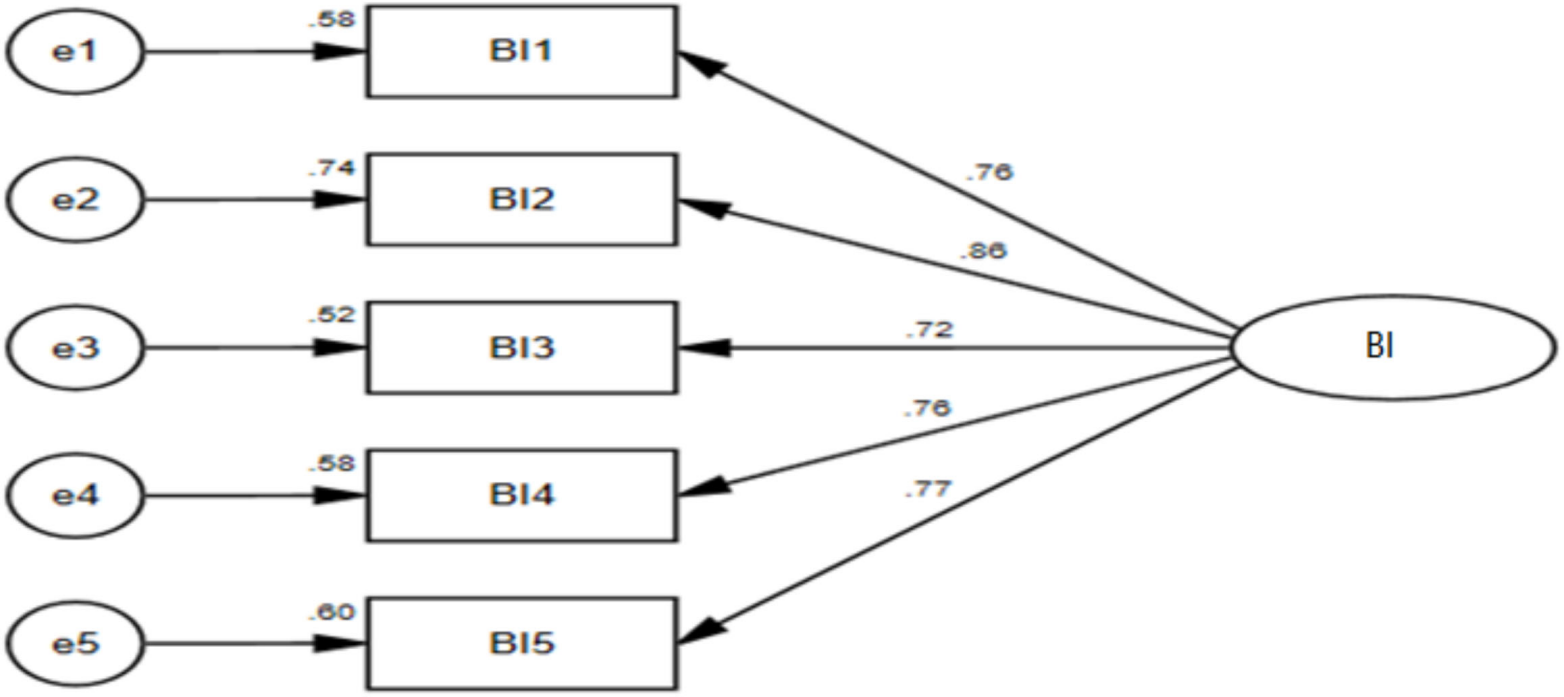
Confirmatory factor analysis diagram of bootleg innovation.

**FIGURE 5:**
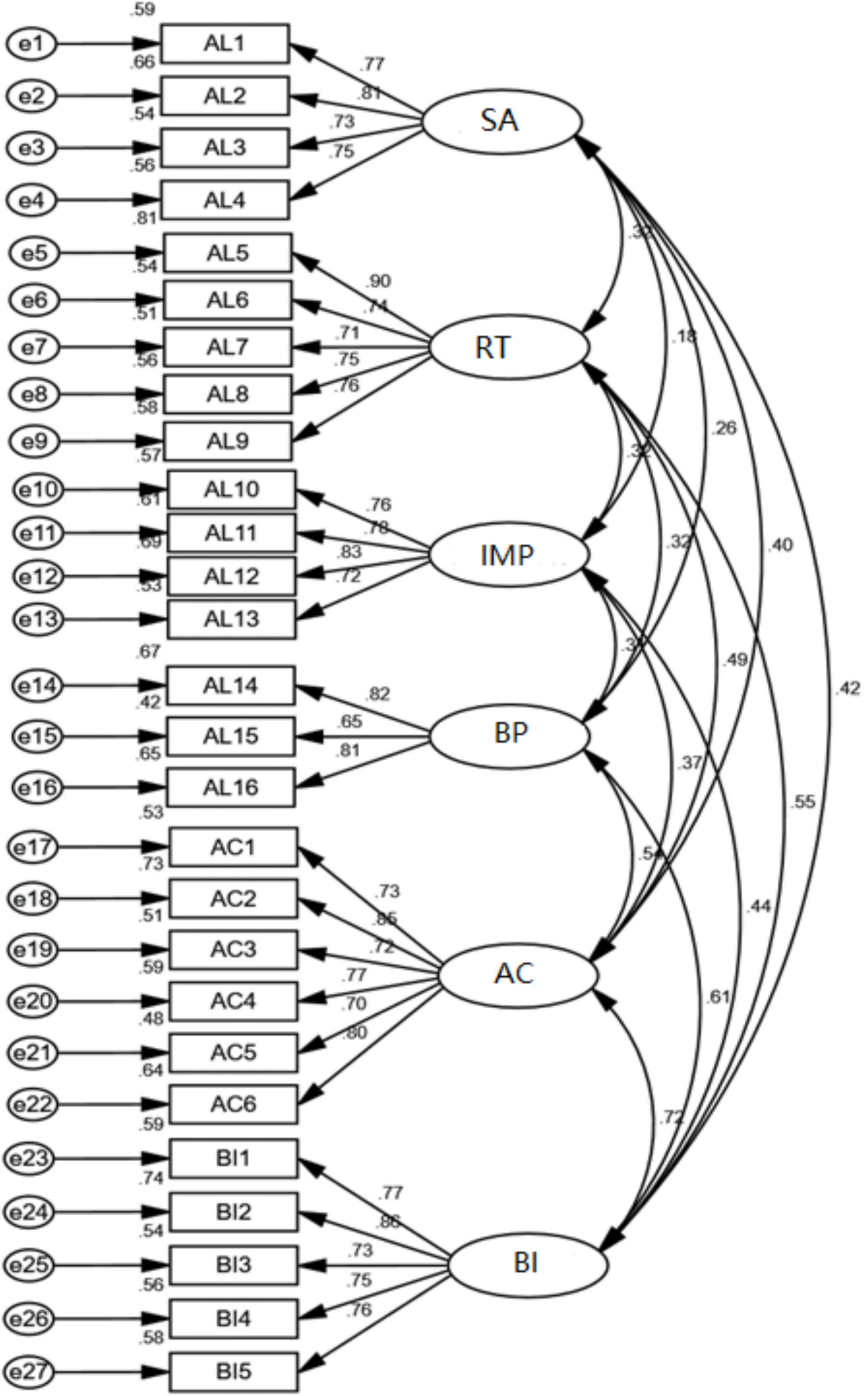
Confirmatory factor analysis model diagram.

In terms of structural validity, It can be seen from Table 10 that χ^2^/df = 1.843 < 3; RMSEA = 0.047 < 0.08; GFI = 0.943 > 0.90; TLI = 0.963 > 0.90; CFI = 0.970 > 0.90. All indexes meet the ideal value, indicating that the model is acceptable. χ^2^/df = 2.480 < 3; RMSEA = 0.063 < 0.08; GFI = 0.982 > 0.90; TLI = 0.981 > 0.90; CFI = 0.988 > 0.90. All indexes meet the ideal value, indicating that the model is acceptable. χ^2^/df = 2.068 < 3; RMSEA = 0.053 < 0.08; GFI = 0.989 > 0.90; TLI = 0.989 > 0.90; CFI = 0.994 > 0.90. All indexes meet the ideal value, indicating that the model is acceptable. χ^2^/df = 1.309 < 3; RMSEA = 0.029 < 0.08; GFI = 0.927 > 0.90; TLI = 0.989 > 0.90; CFI = 0.982 > 0.90. All indexes meet the ideal value, indicating that the model is acceptable. The structure of each variable and the overall structure the following Figures 2–5.

In terms of the validity of the aggregation, The Cronbach’s alpha coefficients of all variables in the reliability and validity test were all above 0.7. The composite reliability (CR) values ranged from 0.804 to 0.941. The factor loadings of each variable item were within the range of 0.645 to 0.803, and the average variance extracted (AVE) values were between 0.507 and 0.550. All these indicators met the corresponding standard values, indicating that the scale has good reliability and convergent validity (Henseler et al., 2015). As illustrated in Table 11, the standardized factor loadings for each question exceed 0.5, and the residuals are both positive and statistically significant, indicating no violations of estimation assumptions. The Composite Reliability (CR) and Average Variance Extracted (AVE) values for self-awareness were found to be 0.849 and 0.585, respectively; for transparent relationships, the CR value was 0.881 with an AVE of 0.599; internalized morality exhibited a CR of 0.856 and an AVE of 0.599; Balanced processing had a CR value of 0.806 alongside an AVE value of 0.582; affective commitment recorded a CR value of 0.892 with an AVE of 0.581; finally, bootleg innovation showed a CR value of 0.883 and an AVE value of 0.602. All observed CR values exceeded the threshold of 0.7, while all AVE values surpassed the minimum requirement of 0.5, thereby satisfying the criteria for convergent validity as well as demonstrating acceptable levels of reliability across constructs examined in this study. Consequently, all items were retained for further analysis.

**TABLE 11:** Confirmatory factor analysis parameter table of each scale.

Item	Path	Variable	Estimate	S.E.	C.R.	*P*	F.L.	CR	AVE
AL1	<—	SA	1.000				0.765	0.849	0.585
AL2	<—	SA	0.981	0.066	14.787	***	0.814		
AL3	<—	SA	0.840	0.060	13.908	***	0.733		
AL4	<—	SA	0.876	0.065	13.427	***	0.746		
AL5	<—	RT	1.000				0.898	0.881	0.599
AL6	<—	RT	0.755	0.045	16.810	***	0.736		
AL7	<—	RT	0.712	0.044	16.020	***	0.713		
AL8	<—	RT	0.832	0.048	17.392	***	0.750		
AL9	<—	RT	0.856	0.048	17.957	***	0.760		
AL10	<—	IMP	1.000				0.755	0.856	0.599
AL11	<—	IMP	1.005	0.072	13.970	***	0.778		
AL12	<—	IMP	1.042	0.066	15.668	***	0.833		
AL13	<—	IMP	1.040	0.078	13.348	***	0.725		
AL14	<—	BP	1.000				0.820	0.806	0.582
AL15	<—	BP	0.770	0.063	12.136	***	0.651		
AL16	<—	BP	1.027	0.071	14.523	***	0.807		
AC1	<—	AC	1.000				0.731	0.892	0.581
AC2	<—	AC	1.162	0.072	16.232	***	0.852		
AC3	<—	AC	1.085	0.080	13.514	***	0.716		
AC4	<—	AC	1.064	0.073	14.549	***	0.768		
AC5	<—	AC	0.954	0.073	13.126	***	0.696		
AC6	<—	AC	1.140	0.075	15.193	***	0.801		
BI1	<—	BI	1.000				0.770	0.883	0.602
BI2	<—	BI	1.198	0.069	17.478	***	0.861		
BI3	<—	BI	0.922	0.063	14.696	***	0.734		
BI4	<—	BI	1.064	0.072	14.831	***	0.747		
BI5	<—	BI	1.038	0.068	15.196	***	0.761		

***P < 0.001.

### Correlation analysis

In the preceding article, the structure of each dimension and its corresponding topic were established through validity and reliability analyses. The average score for each dimension was computed to represent that dimension’s score, followed by a correlation analysis. Correlation analysis primarily investigates the relationships between variables. The range of correlation coefficients spans from −1 to 1; thus, a larger absolute value indicates a stronger relationship between the variables. A detailed classification of correlation coefficients is presented in Table 12.

**TABLE 12:** Correlation degree criteria.

Scope	Degree of relevance
|r| = 1	Perfect correlation
0.70 or less |r| < 0.99	Highly correlated
0.40 ≤ |r| < 0.69	Moderate correlation
0.10 or less |r| < 0.39	Low correlation
|r| < 0.10	Weak or irrelevant

Differential validity analysis aims to determine whether the correlation between two distinct dimensions is statistically significant. Items situated on different isomorphic surfaces should not exhibit high correlations. A correlation coefficient exceeding 0.85 suggests that these items are measuring the same construct, which typically occurs when there is considerable overlap in the definitions of the dimensions involved. In this study, a more rigorous approach utilizing Average Variance Extracted (AVE) has been employed to assess differential validity. Specifically, the square root of the AVE for each factor must exceed the correlation coefficients of all pairwise variable comparisons, thereby indicating that the factors possess differential validity.

Based on the correlation coefficients presented in Table 13, it can be concluded that affective commitment in this study exhibits a significant correlation with self-awareness, relationship transparency, Internalized moral perspective, and Balanced processing among authentic leaders (*p* < 0.01). Additionally, bootleg innovation shows a significant correlation with authentic leaders’ self-awareness, relationship transparency, Internalized moral perspective, and Balanced processing (*p* < 0.01). Moreover, a significant correlation has been identified between affective commitment and bootleg innovation (*p* < 0.01). Moreover, the absolute values of the correlation coefficients are all less than 0.69; both values are also below the square root of the Average Variance Extracted (AVE), indicating that there is not only a no correlation but also an appropriate level of differentiation among all variables. This suggests that the degree of differentiation within the scale is ideal.

**TABLE 13:** Discriminative validity and correlation analysis.

Variable	SA	RT	IMP	BP	AC	BI
SA	0.765					
RT	0.288**	0.774				
IMP	0.156**	0.280**	0.774			
BP	0.211**	0.293**	0.275**	0.763		
AC	0.357**	0.438**	0.321**	0.465**	0.762	
BI	361**	495**	382**	517**	0.641**	0.776
AVE	0.585	0.599	0.599	0.582	0.581	0.602

**P < 0.01.

### Common method variation test

In this study, the Harman single-factor test method is employed to conduct a homogeneity of variance test on the recovered data (Podsakoff and Organ, 1986). All measured data are analyzed using SPSS for unrotated factor analysis to assess the degree of variation attributed to a single factor. The results indicate that there are six factors with eigenvalues greater than 1. However, the variance explained by the first component is 34.401%, which falls below the threshold of 40%. Therefore, it can be concluded that the issue of homologous variance in this study is not significant.

### Structural equation model

**FIGURE 6:**
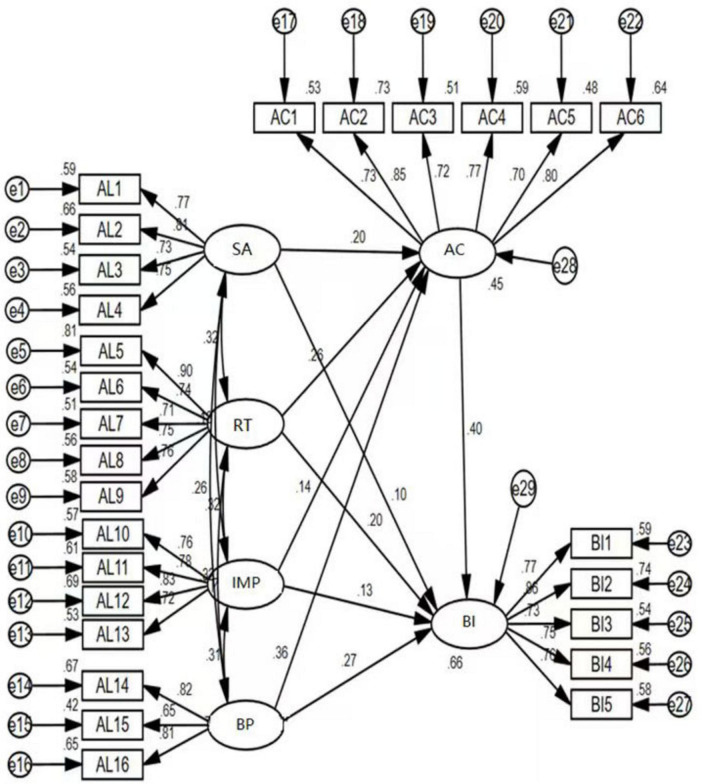
Structural equation model diagram.

AMOS, which stands for “Analysis of Moment Structure,” is a software tool utilized for data analysis in the context of structural equation modeling. The application of AMOS facilitates the examination of covariance structures and enables causal analysis. Building upon the theoretical framework and conceptual model delineating the relationships among authentic leadership, affective commitment, and deviance innovation presented in Chapters 2 and 3, as well as incorporating descriptive statistical analyses, reliability assessments, and validity evaluations conducted on the sample data collected in Chapter 4, this chapter constructs a structural equation model to elucidate the interrelationships among these three variables using AMOS version 24.0. The findings are illustrated in Figure 6.

It can be seen from Table 14 that χ^2^/df = 1.309 < 3; RMSEA = 0.029 < 0.08; CFI = 0.982 > 0.90; NFI = 0.929 > 0.90; TLI = 0.980 > 0.90. They meet the ideal value, indicating that the structural equation model is acceptable.

**TABLE 14:** Fitting degree of structural equation model.

Fitting index	χ^2^	df	χ^2^/df	RMSEA	CFI	NFI	TLI
Standard values			<3	<0.08	>0.90	>0.90	>0.90
Inspection value	404.422	309.000	1.309	0.029	0.982	0.929	0.980

It can be observed from Table 15 that, within the path hypothesis of this study, self-awareness exerts a significant positive influence on affective commitment (β = 0.201, *P* < 0.001). Additionally, relationship transparency also demonstrates a significant positive effect on affective commitment in the context of the path hypothesis (β = 0.264, *P* < 0.001). Furthermore, the internalized moral concept is found to have a significant positive impact on affective commitment (β = 0.136, *P* < 0.01), while Balanced processing similarly shows a substantial positive effect on affective commitment (β = 0.361, *P* < 0.001). Finally, the model accounts for an interpretation of the mediating variable affective commitment at a value of 0.454, representing approximately 45.4%. Authentic leadership has a positive effect on emotional commitment (β = 0.205, *P* < 0.001).

**TABLE 15:** Structural equation model parameter table.

Item	Path	Variable	Estimate	S.E.	C.R.	*P*	Standardized estimates	R square
AC	<—	SA	0.175	0.046	3.806	***	0.201	0.454
AC	<—	RT	0.220	0.046	4.826	***	0.264	
AC	<—	IMP	0.136	0.052	2.594	0.009	0.136	
AC	<—	BP	0.323	0.053	6.063	***	0.361	
BI	<—	SA	0.084	0.038	2.224	0.026	0.101	0.657
BI	<—	RT	0.156	0.038	4.065	***	0.196	
BI	<—	IMP	0.124	0.043	2.865	0.004	0.129	
BI	<—	BP	0.229	0.046	4.965	***	0.268	
BI	<—	AC	0.378	0.059	6.425	***	0.396	

***P < 0.001.

In the path hypothesis, self-awareness demonstrated a significant positive effect on bootleg innovation (β = 0.101, *P* < 0.05). Additionally, relational transparency exhibited a substantial positive influence on bootleg innovation (β = 0.196, *P* < 0.001). Furthermore, internalized morality also had a noteworthy positive impact on bootleg innovation (β = 0.129, *P* < 0.01). Moreover, Balanced processing was found to have a significant positive effect on bootleg innovation (β = 0.268, *P* < 0.001), while the mediating variable of affective commitment showed a considerable positive impact on bootleg innovation as well (β = 0.396, *P* < 0.001). Finally, the R-squared value for the model’s explanation of bootleg innovation through the mediating variable is reported at 0.657, indicating that it accounts for approximately 65.7% of the variance in this construct. Authentic leadership has a significant positive effect on deviant innovation behavior (β = 0.409, *P* < 0.001).

### The test of intermediary effect

Through the structural equation modeling approach, this study preliminarily identified that the independent variables of self-awareness, relationship transparency, internalized moral concepts, and Balanced processing exert significant positive effects on the mediating variable of affective commitment. Furthermore, affective commitment demonstrates a substantial positive impact on bootleg innovation. To assess the significance of the mediating effect, a Bootstrap mediation effect test was conducted. Specifically, Bootstrap Maximum Likelihood (ML) estimation was employed with 5,000 resampling iterations to evaluate the mediation effect results (Preacher and Hayes, 2008). The findings indicated that when the 95% confidence interval included zero, it suggested no mediation; conversely, if zero was not included in the interval, it confirmed the presence of a mediating effect. The detailed results are presented in Table 16.

**TABLE 16:** Test of mediation effect of bootstrap.

Mediation Paths	Effects	Estimates	Standard error	Bias corrected (95%)		
				Lower	Upper	*P*
SA-AC- BI	Direct effect	0.101	0.046	0.012	0.194	0.025
	Indirect effect	0.080	0.026	0.036	0.136	0.000
	Total effect	0.181	0.051	0.080	0.282	0.000
RT-AC- BI	Direct effect	0.196	0.052	0.094	0.295	0.000
	Indirect effect	0.105	0.028	0.058	0.167	0.000
	Total effect	0.301	0.052	0.197	0.398	0.000
IMP - AC - BI	Direct effect	0.129	0.048	0.032	0.220	0.007
	Indirect effect	0.054	0.024	0.012	0.107	0.012
	Total effect	0.183	0.048	0.084	0.276	0.001
BP- AC- BI	Direct effect	0.268	0.059	0.157	0.388	0.000
	Indirect effect	0.143	0.034	0.085	0.220	0.000
	Total effect	0.411	0.056	0.297	0.515	0.000

As illustrated in the Table above, the direct effect of the mediating pathway encompassing self-consciousness, affective commitment, and bootleg innovation in this study is 0.101, with a 95% confidence interval of [0.012, 0.194]. This interval excludes zero and is significant at the 0.05 level, indicating that the direct effect is valid. The mediating effect is measured at 0.080, with a corresponding 95% confidence interval of [0.036, 0.136], which also excludes zero and achieves significance at the 0.001 level; thus, confirming that a partial mediating effect exists. Furthermore, the total effect stands at 0.181 with a 95% confidence interval of [0.080, 0.282], excluding zero as well and demonstrating significance at the 0.001 level; therefore, establishing that the total effect is valid.

In this study, the direct effect of the intermediary pathway characterized by relationship transparency, affective commitment, and bootleg innovation is estimated at 0.196. The 95% confidence interval for this effect is [0.094, 0.295], which excludes zero and is significant at the 0.001 level; thus, we can confirm that a direct effect exists. The mediating effect is calculated to be 0.105, with a corresponding 95% confidence interval of [0.058, 0.167]. This interval also does not include zero and demonstrates significance at the 0.001 level; therefore, we establish that there is a mediating effect present—specifically, it qualifies as a partial mediating effect. Furthermore, the total effect amounts to 0.301 with a 95% confidence interval of [0.197, 0.398]. This range likewise excludes zero and shows significance at the 0.001 level; hence we affirm that the total effect is established as well.

In this study, the direct effect of the mediating path internalizing moral-affection-bootleg innovation is 0.129, and the 95% confidence interval [0.032,0.220], excluding 0, is significant at 0.001 level, and the direct effect is established. The mediating effect is 0.054, 95% confidence interval [0.012,0.107], excluding 0, and it is significant at the level of 0.05, and the mediating effect is established, and it is a partial mediating effect. The total effect is 0.183, 95% confidence interval [0.084,0.276], without 0, and it is significant at 0.001 level, and the total effect is established.

In this study, the direct effect of intermediary path balance information processing - affective commitment - bootleg innovation was 0.268, and 95% confidence interval [0.157,0.388], excluding 0, was significant at 0.001 level, and the direct effect was established. The mediating effect is 0.143, with a 95% confidence interval [0.085,0.220], without 0, and is significant at 0.001 level. The mediating effect is established, and it is a partial mediating effect. The total effect is 0.411, and the 95% confidence interval [0.297,0.515], excluding 0, is significant at 0.001 level, and the total effect is established.

### Summary of hypothesis test results

After the above analysis, the empirical results are summarized in Table 17.

**TABLE 17:** Summary of hypothesis test results.

Hypothesis item	Hypothesis content	Experiment conclusion
H1	The positive correlation between authentic leadership and employees’ bootleg innovation behavior is established.	YES
H1a	It is established that there is a positive correlation between the self-awareness dimension of H1a authentic leadership and employees’ bootleg innovation behavior.	YES
H1b	It is established that there is a positive correlation between the internalized moral concept dimension of H1b authentic leadership and employees’ bootleg innovation behavior.	YES
H1c	It is established that the relationship transparency dimension of H1c authentic leadership is positively correlated with employees’ bootleg innovation behavior.	YES
H1d	It is established that the balance information processing dimension of H1d authentic leadership is positively correlated with employees’ bootleg innovation behavior.	YES
H2	It is established that authentic leadership is positively correlated with affective commitment.	YES
H2a	It is established that authentic leadership’s self-awareness dimension is positively correlated with affective commitment.	YES
H2b	It is established that the internalized moral dimension of H2A authentic leadership is positively correlated with affective commitment.	YES
H2c	The relationship transparency dimension of H2B authentic leadership is positively correlated with affective commitment.	YES
H2d	The balanced information processing dimension of H2d authentic leadership is positively correlated with affective commitment.	YES
H3	The positive correlation between H3 affective commitment and bootleg innovation is established.	YES
H4	It is established that H4 affective commitment plays an intermediary role between authentic leadership and bootleg innovation behavior.	YES

## Research conclusion

Previous studies on employees’ deviant innovation behaviors have mostly focused on the organizational level (Mainemelis, 2010; Globocnik and Salomo, 2015; Wang H. Y. et al., 2019) and the personal characteristic level of employees (Uczuk, 2019; Yang and Li, 2019 (Jaiswal and Dhar, 2015), while the discussion on the influence of leadership style on employees’ deviant innovation is relatively rare. Some scholars have explored the influence of fatalistic leadership (Wang, 2019), transformational leadership (Wang H. Y. et al., 2019), etc. on employees’ deviant innovation behaviors. Moreover, most of the existing research on true leadership focuses on its cognitive impact, lacking attention to cognitive-emotional aspects (Wu S. J. et al., 2020; Wang and Zhang, 2019). This study employs the social exchange theory and the cognitive-affective personality system theory to construct a mediation model in which authentic leadership exerts emotional commitment on employees’ deviant innovative behaviors based on the Chinese management context, in order to reveal the transmission mechanism of the influence of authentic leadership on employees’ deviant innovative behaviors in the Chinese cultural context. The intermediary variable of affective commitment is introduced to elucidate the potential interrelationships among authentic leadership (encompassing four dimensions: self-awareness, relational transparency, Balanced processing, and Internalized moral perspective), affective commitment, and employees’ bootleg innovation behaviors. Consequently, research hypotheses and a theoretical model are proposed. A total of 378 valid samples from high-tech enterprises were collected. Statistical analyses were conducted using SPSS 22.0 and AMOS 24.0 software to test the hypotheses, yielding the following results.

## Authentic leadership has a positive effect on bootleg innovation

Authentic leadership is a positive and open leadership style that better aligns with the essence of a positive leadership style and helps stimulate employees’ innovative behaviors. This study confirmed that the authentic leadership style can significantly and positively stimulate employees’ deviant innovation behaviors, which is consistent with the previous research conclusions of scholars on the impact of authentic leadership on deviant innovation (Wu S. J. et al., 2020; Wang and Zou, 2019). Authentic leaders possess a strong moral sentiment and a heightened sense of self-restraint, enabling them to have a clear understanding of both themselves and their surrounding environment. They are capable of objectively analyzing relevant information about themselves and their employees, which allows them to effectively facilitate mutual development for both parties while promoting organizational adaptability in response to environmental changes. The integration of high altruism with a robust sense of responsibility fosters a positive and innovative atmosphere within the organization, thereby creating opportunities for employees to engage in innovation and progress. On this foundation, authentic leaders exert influence over their employees, impacting their behaviors significantly. The authenticity exhibited by leaders, along with other characteristics they embody, serves as an exemplary model for employees. Under the sway of these traits, employees are more likely to embrace authentic ideas that can infuse innovative energy into the organization. Even when certain innovative concepts lack organizational support, such influences can stimulate employee potential and encourage active participation in the innovation process—ultimately enhancing their capacity for bootleg innovation. Moreover, when authentic leaders cultivate equitable and transparent relationships with their employees, it facilitates the establishment of effective communication mechanisms between leadership and staff. This interactive dynamic enhances employees’ feelings of trust and security within the organization. Consequently, leaders’ perceptions regarding their workforce can subtly shape attitudes; they may internalize organizational objectives as shared goals among all team members. This alignment makes employees more inclined to contribute positively toward organizational performance while fostering greater commitment toward returning value to the organization itself. Furthermore, it encourages them to prioritize outcomes derived from innovation rather than focusing solely on methods employed during the innovation process—thereby further stimulating deviant innovative behaviors among staff members.

## Authentic leadership has a positive effect on effective commitment

Affective commitment refers to the identification with and emotional attachment to an organization, which can be appropriately cultivated. The principles of social exchange and reciprocity suggest that when employees perceive trust and support from their leaders, they are motivated to engage in beneficial reciprocal behaviors. Therefore, when employees sense the authenticity of their leaders, they become more emotionally invested. Authentic leadership is characterized by consistency between words and actions; it consciously demonstrates positive attitudes such as hope, confidence, openness, optimism, and trust toward employees while embodying the organization’s values through its positive state. Employees are influenced by this upward value system conveyed by their leaders, leading to increased recognition of organizational values and subsequently enhancing affective commitment. Based on the previous research results of this article, it can be seen that genuine leadership has a positive effect on emotional commitment. Some existing scholars’ research has also proved that leadership behavior has a positive impact on employees’ organizational (emotional) commitment, such as calm leadership (Li J. et al., 2016), shared leadership (Park and Seo, 2019), and transformational leadership (Liu and Kong, 2020). In interactions with subordinates, authentic leadership reflects relational transparency and fosters a high-quality environment for sincere communication that enhances employees’ psychological safety. As a result, employees feel comfortable psychologically; they perceive themselves as recognized and treated equally by their leaders. This leads them to reciprocate with higher levels of affective commitment. Moreover, authentic leaders advocate for employee participation in organizational decision-making processes while balancing all relevant information. This approach helps increase employee engagement as individuals feel like integral members of the organization—thereby strengthening their affective commitment. Additionally, authentic leadership pays attention to employees’ personal development and emotional needs; in turn, this focus encourages greater commitment and identification with the organization among staff members. These characteristics of authentic leadership and their influence on emotional commitment are also highly consistent with the scholars’ exploration of the influence mechanism of authentic leadership on emotional commitment (Han and Liu, 2020; Wang and Zhang, 2019).

## Affective commitment has a positive effect on bootleg innovation

Through empirical analysis, it has been found that affective commitment significantly positively influences bootleg innovation. In other words, higher levels of affective commitment can stimulate employees’ abilities for bootleg innovation. Previous research conclusions have also confirmed that emotional commitment has a significant impact on innovative behavior (Ma and Su, 2020; (Leroy et al., 2012), especially with the popularity of remote work after the pandemic, if some scholars have verified that remote employees with high organizational commitment have a stronger positive impact on deviant innovative behavior in terms of their work autonomy (Xiao, 2020). The results of this study are basically consistent with previous studies, but they are also contrary to the research results of some foreign scholars. For example; This finding contrasts with the results of Tenzer and Yang (2020) study, which suggested that increased organizational commitment among employees leads to a decrease in creative deviant behavior. The potential reasons for this discrepancy may lie in the differences between foreign and domestic working environments, as well as variations in emotional concepts between foreigners and Chinese individuals. In foreign workplace settings, while employees with high affective commitment are motivated to generate new ideas aimed at enhancing organizational effectiveness, they tend to be reluctant to engage in creative behaviors that could position them as outliers against their organization (Tenzer and Yang, 2018). Consequently, these individuals often cease pursuing their innovative ideas when leaders instruct them to abandon such pursuits. Conversely, within the Chinese workplace environment, although employees with a high affective commitment rarely confront their leaders directly regarding rejected ideas, they typically choose to accept outcomes silently if their proposals are not embraced. However, when they still perceive their innovative ideas as viable under these circumstances, such employees may resort to bootleg innovation. Moreover, compared to foreign contexts where individual interests might take precedence over collective ones, China places greater emphasis on collective interests. Employees exhibiting high affective commitment abroad may believe that their leaders’ decisions are correct and subsequently relinquish their innovative concepts. In contrast, in China’s context—where organizational goals are viewed as paramount—employees with strong affective commitments often continue demonstrating creative behaviors privately even after having had their innovative suggestions dismissed by leadership; thus, engaging in what is termed “bootleg innovation.”

## Affective commitment plays an intermediary role between authentic leadership and bootleg innovation

At present, empirical research exploring the mediating mechanism of emotional commitment between authentic leadership and deviant innovation is still lacking. However, some scholars have confirmed the mediating role of emotional commitment between authentic leadership and other aspects of employees (Ribeiro et al., 2020; Li et al., 2014). Through research on the intermediary role between authentic leadership and bootleg innovation, it has been identified that affective commitment serves as an effective internal mechanism for interpreting the relationship between these two constructs. Authentic leadership emphasizes employee development, employing both verbal guidance and practical examples. It establishes a compelling organizational vision that enables employees to internalize this vision into their personal goals, fostering trust and identification with the organization while enhancing their affective commitment. The transparency and trust inherent in authentic leadership contribute to creating a positive working atmosphere for employees. In such an environment, employees experience greater job satisfaction over time, leading to increased emotional attachment to the organization. The positive emotions associated with happiness can also expand individuals’ creative thinking capabilities. Furthermore, employees who possess a strong affective commitment to the organization are more likely to proactively seek solutions to work-related challenges and effectively navigate obstacles they encounter.

And they’re more emboldened to take the risk of deviating from the path of innovation. On the contrary, people with weak organizational emotions will avoid things, be unwilling to take risks at work, and will not make private efforts for the benefit of the organization, which will produce relatively big obstacles to bootleg innovation. Authentic leaders can further influence employees’ bootleg innovation behavior by influencing their affective commitment. This is also in line with reality. Employees must first recognize the organization and have a certain emotion for the organization. Only then will they be willing to offer new ideas and solutions to help the organization achieve greater benefits regardless of all risks. If an enterprise only guides employees to deviate and innovate through authentic leadership, the effect will be greatly reduced.

## Practical implications

### Pay attention to and properly use bootleg innovation behavior

Enterprises should recognize that resources are finite and cannot accommodate the innovative ideas of all employees. Given this premise, the emergence of bootleg innovation is an inevitable trend. Organizations ought to acknowledge and effectively leverage bootleg innovation for their benefit, rather than indulging in or arbitrarily restricting it. On one hand, leaders must consistently monitor employee behaviors. When they observe instances of deviant innovative behavior among employees, they should refrain from either unconditionally supporting or opposing such actions. Provided that these behaviors do not disrupt normal work processes, enterprises can afford to relax certain standards and adopt a more tolerant stance toward ongoing bootleg innovations. Concurrently, leaders should assess where organizational rules may be inadequate and make necessary improvements or encourage employees to pursue innovation through formal channels. On the other hand, organizations should establish a robust mechanism for evaluating innovative ideas while enhancing communication with employees whose proposals have not been accepted. It is essential to communicate the reasons behind these decisions to foster understanding among employees and mitigate any negative sentiments toward the organization that could lead to internal discord.

## Focus on the positive role of authentic leadership

### Enterprises should pay attention to the cultivation and development of authentic leaders

The analysis results indicate that authentic leadership exerts a significant positive influence on employees’ bootleg innovation behavior. Consequently, enterprises must focus on the cultivation and development of authentic leadership, as this will enhance employees’ affective commitment and stimulate their bootleg innovation behaviors. In the context of external recruitment and internal selection, organizations should consider leadership characteristics as a critical screening criterion. Attention should be given to whether leaders demonstrate consistency between their words and actions, treat employees with honesty and equity, establish transparent and genuine relationships with staff members, foster mutual positive feedback, and create synergies that exceed individual contributions. Organizations are encouraged to prioritize candidates who exhibit authentic leadership traits. Furthermore, in the training programs for leading cadres, there should be a strong emphasis on nurturing authentic leaders.

### The leader’s behavior should be closer to the real leader

Leaders should be mindful of the connotations associated with authentic leadership, continuously striving to enhance their values and guiding their teams through these positive principles. An authentic leader ought to consistently use the concept of authentic leadership as a reflective tool to assess their behaviors, engage in ongoing self-improvement, and serve as a role model for others. Simultaneously, leaders must embody authenticity and approachability by actively communicating with employees and endeavoring to establish trusting relationships that foster employee recognition and engagement. Finally, leaders should maintain impartiality when addressing information provided by employees, ensuring an objective evaluation process. This approach cultivates an atmosphere of equality within the workplace, thereby enabling employees to leverage their innovative capabilities more effectively.

## Improve the value fit between employees and the organization, and cultivate employees’ affective commitment to the organization

The research findings indicate that affective commitment significantly positively influences bootleg innovation. Consequently, enterprises should prioritize the cultivation of employees’ affective commitment. Affective commitment is fundamentally rooted in the recognition of organizational values. Therefore, enhancing an employee’s value alignment with the organization can be achieved through several strategies. Firstly, organizations can select candidates who exhibit a high level of alignment with their values before they enter into the company. During talent recruitment interviews, it is essential not only to assess applicants’ professional competencies but also to evaluate the degree of congruence between their values and those of the enterprise. Secondly, once employees have joined the organization, training programs can be implemented to foster a stronger sense of identification with corporate values. Additionally, providing humane care for employees can further enhance their affective commitment to the company.

## Limitations and future research

Based on the social exchange theory and the cognitive-affective personality system theory, this study investigates the relationship between authentic leadership, affective commitment, and employees’ bootleg innovation behavior. Employing a scientific questionnaire survey and data analysis methods, the research hypotheses and theoretical model are empirically tested, yielding valuable findings. However, due to inherent limitations within the study’s conditions, certain deficiencies remain that warrant further improvement.

## The sample scope and level can be appropriately expanded

First and foremost, due to the limitations of my abilities and resources, the sample enterprises are exclusively drawn from Beijing, Shenzhen, and more developed regions of Zhejiang, such as Hangzhou and Ningbo. This selection may lack a certain degree of generalizability. Secondly, I did not comprehensively consider all types of enterprises; the findings of this study focus solely on high-tech sectors including IT, biomedicine, machinery manufacturing, and other industries that require innovative achievements. Consequently, the results may lack comprehensiveness. Finally, it is important to note that all questionnaires in this study were based on self-evaluations by enterprise employees. This approach may be subject to homology error to some extent. In light of these considerations regarding sample scope and methodology: future research could expand its investigation to include a broader range of regions and industries. Additionally, at the questionnaire level, subsequent studies might employ a paired approach involving both leaders and employees for data collection. This would facilitate a deeper understanding of the relationships among the three variables involved and enable more scientifically robust conclusions.

## Adjust the model: find other mediating variables or add moderating variables

This study focused exclusively on affective commitment as a mediating variable between authentic leadership and employees’ bootleg innovation behavior. However, there are additional potential mediators to consider, such as self-efficacy, moral identity, and other relevant factors. Future research could explore these alternative mediating variables to further enhance the model. Moreover, this study did not examine the moderating variables that may influence the relationship between authentic leadership and employees’ bootleg innovation behavior. Potential moderating factors, such as uncertainty avoidance, warrant investigation. Subsequent studies should consider incorporating various moderating variables to broaden the understanding of their effects in this context.

## Future research could investigate the potential negative impacts of deviant innovative behaviors

In the future outlook section, the negative impacts of deviant innovation on organizations have been supplemented. This study has found that authentic leadership has a positive effect on deviant innovation behavior, which is consistent with the conclusions of many previous scholars. Deviant innovation, as a behavior with dual attributes of “legitimate purpose” and “illegitimate means” (Huang et al., 2017), inherently implies the duality of its consequences. However, the academic community has explored its negative impacts very little. Only a few studies have pointed out that deviant innovation behavior can lower the expectations of organizational members for management norms, be detrimental to the management’s control over the R&D process, and even trigger counterproductive behaviors among organizational members, thereby reducing organizational performance (Lin et al., 2016). Additionally, Wang H. Y. et al. (2019) suggest that in mature teams, when members with lower informal status successfully engage in deviant innovation, it can severely undermine organizational cohesion. The lack of attention to the negative impacts of deviant innovation can mislead organizations into overemphasizing its positive effects and prevent them from forming an objective evaluation. Therefore, future research could specifically explore the negative impacts of deviant innovation on employees themselves, leaders, and colleagues.

## Data Availability

The original contributions presented in this study are included in this article/supplementary material, further inquiries can be directed to the corresponding author.
